# Astrocytes acquire morphological and functional characteristics of ependymal cells following disruption of ependyma in hydrocephalus

**DOI:** 10.1007/s00401-012-0992-6

**Published:** 2012-05-11

**Authors:** Ruth Roales-Buján, Patricia Páez, Montserrat Guerra, Sara Rodríguez, Karin Vío, Ailec Ho-Plagaro, María García-Bonilla, Luis-Manuel Rodríguez-Pérez, María-Dolores Domínguez-Pinos, Esteban-Martín Rodríguez, José-Manuel Pérez-Fígares, Antonio-Jesús Jiménez

**Affiliations:** 1 Departamento de Biología Celular, Genética y Fisiología, Universidad de Málaga, Campus Universitario de Teatinos, 29071 Málaga, Spain; 2 Instituto de Anatomía, Histología y Patología, Facultad de Medicina, Universidad Austral de Chile, Valdivia, Chile

**Keywords:** Cerebrospinal fluid, Congenital hydrocephalus, Ependyma disruption, Astrocyte reaction, Barrier properties, Permeability, Transport, hyh mice, Human

## Abstract

Hydrocephalic hyh mutant mice undergo a programmed loss of the neuroepithelium/ependyma followed by a reaction of periventricular astrocytes, which form a new cell layer covering the denuded ventricular surface. We present a comparative morphological and functional study of the newly formed layer of astrocytes and the multiciliated ependyma of hyh mice. Transmission electron microscopy, immunocytochemistry for junction proteins (N-cadherin, connexin 43) and proteins involved in permeability (aquaporin 4) and endocytosis (caveolin-1, EEA1) were used. Horseradish peroxidase (HRP) and lanthanum nitrate were used to trace the intracellular and paracellular transport routes. The astrocyte layer shares several cytological features with the normal multiciliated ependyma, such as numerous microvilli projected into the ventricle, extensive cell–cell interdigitations and connexin 43-based gap junctions, suggesting that these astrocytes are coupled to play an unknown function as a cell layer. The ependyma and the astrocyte layers also share transport properties: (1) high expression of aquaporin 4, caveolin-1 and the endosome marker EEA1; (2) internalization into endocytic vesicles and early endosomes of HRP injected into the ventricle; (3) and a similar paracellular route of molecules moving between CSF, the subependymal neuropile and the pericapillary space, as shown by lanthanum nitrate and HRP. A parallel analysis performed in human hydrocephalic foetuses indicated that a similar phenomenon would occur in humans. We suggest that in foetal-onset hydrocephalus, the astrocyte assembly at the denuded ventricular walls functions as a CSF–brain barrier involved in water and solute transport, thus contributing to re-establish lost functions at the brain parenchyma–CSF interphase.

**Electronic supplementary material:**

The online version of this article (doi:10.1007/s00401-012-0992-6) contains supplementary material, which is available to authorized users.

## Introduction

Congenital hydrocephalus is a developmental brain disorder. In the humans, its incidence is approximately 1–3 in every 1,000 live births. Dilatation of the brain ventricles and elevation of intraventricular pressure in rats with congenital or acquired hydrocephalus have harmful effects on the parenchyma and lead to oedema, oxidative stress [57], proteolytic damages in the white matter [15], cell death and reactive changes in glial cells [17]. Some of these alterations have also been reported in human chronic hydrocephalus [16]. Under these abnormal brain conditions, some mechanisms can be triggered to partially re-establish brain homoeostasis [31].

Astroglial reactions triggered by brain injuries are characterized by the hypertrophy and hyperplasia of astroglial cells. Astrocyte reactions have been reported to inhibit axonal regeneration [7], but have also been associated with the secretion of growth factors and trophic molecules [19, 24, 47, 49] such as NGF, IGF-I and bFGF that promote axonal re-growth [47]. It has been suggested that the astrocyte reaction initially protects the brain tissue and contributes to its functional recovery [22]. Therefore, the beneficial and detrimental functional consequences of these astroglial reactions are under debate [36, 58, 59]. The astrocyte reaction that occurs in the brain of hydrocephalic animals has been thought to be a harmful phenomenon, leading some authors to test the effects of anti-inflammatory drugs in rats that have been made hydrocephalic postnatally [35, 36].

There is a large body of evidence indicating that the neuroepithelium/ependyma lining the ventricular walls of the developing brain plays a key role in the onset and evolution of congenital hydrocephalus [1–3, 18, 26, 52, 55, 61, 65]. There are several ependymal cell lineages lining distinct regions of the ventricular walls. In the cerebral aqueduct of hydrocephalic hyh mice, different types of ependymal cells have been reported [65]. Some of these types disrupt, some remain unaffected and other ones proliferate [4, 65]. Recently, different types of ependymal cells have been described in the cerebral aqueduct of human foetuses [55]. Ependymal specializations also occur in other regions of the ventricular system [50]. Most of the different ependymal populations are multiciliated. Their cilia beat in a synchronized manner and thus contribute to the flow of the cerebrospinal fluid (CSF) [43, 68, 69]. Other functions have also been assigned to the multiciliated ependyma, including the regulation of the interaction between the ventricular CSF and the brain extracellular fluid, the clearance of metabolic substances and neurotransmitters and the mediation of adhesion of inflammatory cells [14, 65]. We have previously reported that foetal-onset hydrocephalus in mutant hyh mice and in human foetuses is associated with defects in the neuroepithelium/ependyma [18, 30, 43]. Such defects result in the loss of the neuroepithelium/ependyma and in a subsequent astroglial reaction that leads to the development of a new cell layer lining the denuded ventricular surface [18, 41, 65]. The functional significance of the new brain parenchyma/CSF interphase formed by reactive astrocytes is not known.

The present study has been designed to help elucidate the role of the periventricular astroglial reaction in congenital hydrocephalus. The study was performed on hyh (*hy*drocephalus with *h*op gait) [11] mutant mice, in which the onset and evolution of hydrocephalus resemble that of human congenital hydrocephalus. Two phases have been recognized in the development of congenital hydrocephalus in the hyh mutant mouse. During embryonic life, the neuroependyma disruption of the ventral fourth ventricle and cerebral aqueduct is followed by a moderate communicating hydrocephalus. During the first postnatal week, the dorsal wall of the cerebral aqueduct becomes disrupted triggering aqueduct obliteration and the onset of a severe hydrocephalus [65]. The investigation was carried out at the postnatal stages when hydrocephalus is severe and astrogliogenesis and astrocyte reaction are completed [41]. The study also analysed the astrocyte reaction occurring in the denuded ventricular walls of human hydrocephalic foetuses.

## Materials and methods

### Animals

Mutant hyh mice (hydrocephalus with hop gait, B6C3Fe-a/a-hyh/J strain) and their control littermate wild-type (wt) mice were used [11]. The hyh mouse carries a point mutation in the *Napa* gene that encodes α-Snap [13, 25], a protein involved in membrane fusion events. Mice were obtained from The Jackson Laboratory (Bar Harbor, ME, USA) and bred into two colonies, one at the Animal Experimentation Service of the University of Malaga and the other at the Medical School of the Austral University of Chile, Valdivia, Chile. The housing, handling, care and processing of the animals were conducted in accordance with the European and Spanish laws (DC 86/609/CEE and RD 1201/2005) and following the regulations approved by the council of the American Physiological Society. Wt and mutant hyh mice were identified by clinical inspection and genotyping [5]. The animals were anesthetized with intraperitoneally administered Dolethal (sodium pentobarbital; Vétoquinol, Lure, France; 0.2 mg/g bodyweight) and killed at the postnatal (P) ages detailed in Table 1.

**Table 1:** Number of animals by age and genotype used in each experiment

Experiment	Immunocytochemistry	Immunofluorescence	HRP tracing (light microscopy)	HRP tracing (electron microscopy)	Lanthanum tracing	Scanning electron microscopy
Animal postnatal age in days (number/genotype)	10, 14, 20, 30 (4 wt and 4 hyh each age)	6 (6 wt, 6 hyh), 14 (6 wt, 6 hyh), 20 (7 wt, 7 hyh), 30 (3 wt, 3 hyh)	3, 8, 14, 30 (2 wt and 4 hyh each age)	3 (2 each condition), 30 (2 wt, 4 hyh)	3 (3 wt, 5 hyh), 6 (3 wt, 5 hyh), 10 (3 wt, 5 hyh), 20 (5 wt, 5 hyh), 30 (3 wt, 5 hyh)	14 (6 hyh), 20 (10 hyh)

### Human foetuses

Paraffin sections of brains from 8 foetuses presenting communicating hydrocephalus and 15 control foetuses were used (for further information concerning this material see [18]).

### Immunocytochemistry

Wt and hyh mice were transcardially perfused with Bouin fixative. The brain was dissected out and further fixed by immersion in Bouin fixative for 2 days. Serial paraffin sections were obtained and adjacent sections were incubated with a series of primary antibodies (Table 2). For further details, see Supplementary data.

**Table 2:** Primary antibodies used

Antibody (reference)	Source	Type	Dilution	Molecule/structure labelling
Aquaporin 4 (A5971)	Sigma	Rabbit polyclonal	1:400	Water channel protein
Caveolin-1 (N-20)	Santa Cruz Biotechnology, INC, San Diego, CA, USA	Rabbit polyclonal	1:200	Caveolae
Connexin 43	^a^	Rabbit polyclonal	1:750	Gap junctions
EEA1 (PA1-063)	Affinity Bioreagents INC, Gonden, CO, USA	Rabbit polyclonal	1:200	Early endosome antigen 1
GFAP (4650)	Biogenesis, Oxford, UK	Rabbit polyclonal	1:250	GFAP intermediate filaments
GFAP (4674)	Abcam, Cambridge, UK	Mouse monoclonal	1:1,000	GFAP intermediate filaments
GLUT1	^b^	Rabbit polyclonal	1:1,000	Glucose transporter 1
HRP	^c^	Rabbit polyclonal	1:1,000	Injected HRP
N-Cadherin (sc-8939)	Santa Cruz Biotechnology	Rabbit polyclonal	1:50	N-Cadherin (adherens junctions)
S100β (ab52642)	Abcam	Rabbit polyclonal	1:200	S100β
TGN46 (ab16059)	Abcam	Rabbit polyclonal	1:500	Trans-Golgi network
Tubulin βIV (T7941)	Sigma	Mouse monoclonal	1:400	Ependymal cilia
Tubulin βIV (ab11315)	Abcam	Mouse monoclonal	1:100	Ependymal cilia
Vimentin (V4630)	Sigma, St Louis, MO, USA	Goat polyclonal	1:500	Vimentin intermediate filaments

^a^Kindly provided by JC Sáez, Catholic University of Chile

^b^Kindly provided by CI Ribas, Memorial Sloan-Kettering Cancer Center, NY, USA

^c^Developed at the Department of Cell Biology, Genetics and Physiology, University of Malaga, Spain

### Single and double immunofluorescence and confocal microscopy

Wt and hyh mice (Table 1) were transcardially perfused with Bouin fixative or 4 % paraformaldehyde diluted in 0.1 M phosphate buffer (PB), pH 7.4. Bouin-fixed brains from P6 (6 days of age) and P30 mice were used to obtain paraffin sections that were hydrated and immunostained. Paraformaldehyde-fixed brains from P14 and P20 mice were used to obtain frozen sections that were immunostained with a free-floating section-staining protocol. In four P20 mice, the ventricular walls of the lateral ventricles were dissected out to obtain whole mounts for immunostaining. After incubation in the primary antibody (Table 2), appropriate fluorescent secondary antibodies were used.

Adjacent sections from the series obtained from the brain of P6, P20 and P30 wt and hyh mice, and whole mounts from the lateral ventricles of P14 and P20 hyh mice were processed for double immunofluorescence. This procedure allowed the same brain regions to be analysed with a series of antibodies. For further details, see the Supplementary data.

### Intracerebroventricular injections of horseradish peroxidase

Wt and hyh mice (Table 1) were anesthetized with 2,2,2-tribromoethanol (Sigma, 0.8 μg/g body weight). Wt and hyh mice were subperfused into the left lateral ventricle for 5 min with 1 μl (P3), 1.5 μl (P8), 2 μl (P14) and 2.5 μl (P30) of 3 % horseradish peroxidase (HRP) type IV (Sigma) in 0.9 % sodium chloride. The coordinates for injection in wt and hyh mice at different ages were previously calculated using injections with trypan blue. After the infusion, the needle remained at the injection site for an additional 15 min. The brains were processed to trace HRP at the light microscope. The brains were dissected out, fixed by immersion with Bouin fixative for 72 h, embedded in paraffin and serially sectioned. The sections were processed for the immunoperoxidase method using an anti-HRP antibody raised in rabbit in our laboratory. Adjacent sections were immunostained for GFAP.

To trace HRP with the electron microscope, wt and hyh mice at P3 and P30 (Table 1) were injected with the tracer into the left lateral ventricle, as described above. Five minutes after the injection, the animals were transcardially perfused with phosphate buffer containing 2 % paraformaldehyde and 2.5 % glutaraldehyde. Vibratome sections, 50-μm thick, were obtained and processed for the histochemical detection of HRP using DAB as the electron donor. The sections were postfixed in 1 % osmium tetroxide (Merck, Darmstadt, Germany) and flat embedded in Araldite 502. Ultrathin sections (60-nm thickness) were stained with lead citrate and studied under an electron microscope.

### Lanthanum nitrate tracing at the electron microscope

To demonstrate the presence or absence of tight junctions in the ependymal and astrocyte barriers, lanthanum nitrate was used as a tracer under electron microscope [39, 48]. Wt and hyh mice (Table 1) were anesthetized and transcardially perfused with 2.5 % glutaraldehyde in 0.1 M cacodylate buffer, pH 7.4, and the brains were dissected out and immersed in fresh fixative for 1 h. After fixation, 2 % lanthanum nitrate in cacodylate buffer, pH 7.8, was delivered over 2 min into one of the lateral ventricles, the cerebral aqueduct or the fourth ventricle. The brains were further fixed in fresh fixative for 24 h at 4 °C. The walls of the cerebral aqueduct, the lateral, third and fourth ventricles, and the choroid plexus were dissected out and postfixed in 1 % osmium tetroxide in cacodylate buffer for 1 h at 4 °C. The tissue blocks were dehydrated and embedded in Araldite 502 (EMS, Hatfield, PA, USA). Ultrathin sections (60-nm thickness) were slightly stained with uranyl acetate and studied under an electron microscope (Philips CM100). The blood–brain barrier at the choroid plexus and the endothelial cells of the brain capillaries (presence of tight junctions) were used as controls.

### Scanning electron microscopy

Killed hyh mice (Table 1) were used. Cold 2.5 % glutaraldehyde in phosphate buffer was injected into a lateral ventricle for 2 min. The brains were further fixed by immersion in the same fixative for 2 h at room temperature. Several areas of the ventricular cavities were dissected out and processed as previously described [30].

### Data analysis

Morphometric, densitometric and image analyses were carried out to quantify several parameters. (1) The relative optic density of aquaporin 4 immunoreaction in ependymal cells, astrocyte cell bodies and perivascular endfeet of astrocytes was estimated. (2) The total area occupied by the early endosomal compartment (EEA-1 immunoreactivity) in ependymal cells or astrocytes was quantified. (3) The cell density of GFAP+ astrocytes at specific sites of the abnormal ventricles was estimated using whole mounts of ventricular walls of four hyh P20 mice processed for immunofluorescence for GFAP. (4) The degree of penetration of intraventricularly injected HRP into the brain parenchyma was estimated in sections immunostained using anti-HRP. See Supplementary data for a description of the procedures.

## Results

In the description that follows, the morphological phenotype and the barrier properties of the multiciliated ependyma of wt mice will be compared with those of the multiciliated ependyma of hyh mice resisting denudation [41] and, of particular interest for the present study, with those of the astrocyte layer covering the denuded ventricular regions of the hyh mice. Most of this study were carried out at stages from P6 on, when the denudation process and the astrocyte reaction are completed [41] and a severe progressive hydrocephalus develops (Supplementary Fig. 1). After P6, most of the ventricular surface, with the exception of some specific sites, underwent denudation (Supplementary Fig. 2). The denudation sites resisting denudation are the circumventricular organs, the roof of the third ventricle, the roof of the middle region of the cerebral aqueduct and small patches consistently present at very specific sites of the aqueduct and lateral ventricles (Supplementary Fig. 2). All denuded areas presented periventricular astrocyte reactions. However, the description of the results will principally circumscribe to events occurring in the fourth and lateral ventricles. In hyh mice, the lateral ventricles are enormously expanded, whereas the fourth ventricle is not. However, both cavities undergo ependymal denudation and astrocyte reaction, indicating that such processes are associated with the genetic defect of these mice and not with the ventriculomegaly or increased intraventricular pressure. Therefore, it seemed relevant to compare the properties of the astrocytes lining the denuded areas in the fourth and lateral ventricles.

### Cytoskeletal proteins in multiciliated ependyma and astrocytes covering the ventricular walls of wt and hyh mice

**Fig. 1:**
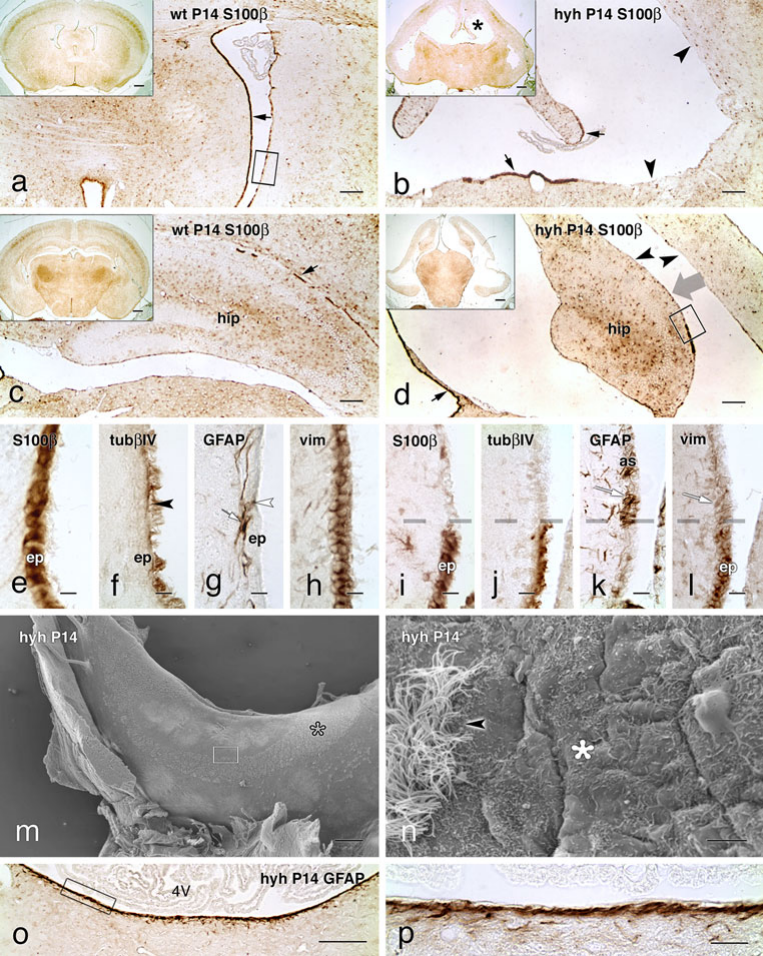
Expression of S100β and cytoskeleton proteins in the ependymal cells and astrocytes covering the ventricular surface of wt and hyh mice at P14. Frontal sections of the telencephalon and fourth ventricle (4V) of wt (**a**, **c**, **e**–**h**) and hyh (**b**, **d**, **i**–**l**, **o**, **p**) mice immunostained for S100β protein, tubulin βIV (tubβIV), GFAP and vimentin (vim). **a**–**d** The ependyma (*ep*) contains S100β protein (*black arrows*). In the hyh mice, the lateral ventricles are enlarged (*black* *asterisk*) and most of their surface is devoid of ependyma (*black arrowheads*). The *thick grey arrow* in **d** indicates the direction at which the area framed is shown under the scanning microscope as shown below in this panel. **e**–**h** Adjacent sections of an area similar to that framed in **a**. TubβIV is present in ependymal cilia (*black* *arrowhead* in **f**). In the wt mouse, GFAP+ astrocytes (*as*) lying in the subependymal (*white arrow*; in **g**) and ependymal (*white arrowhead*; in **g**) region are observed. **i**–**l** Adjacent sections of an area similar to that framed in **d** bordering the hippocampus. The *broken line* in **i**–**l** denotes the border between an ependymal patch resisting denudation (*bottom*) and the astrocyte layer covering the denuded surface (*top*). Reactive astrocytes are GFAP+ and vim+ (*white arrows*; in **k**, **l**). **m**, **n** Scanning electron microscopy of the hippocampus surface overlooking the lateral ventricle (for orientation, see *thick arrow* in **d**). A patch of ciliated ependyma partially covers the ventricle surface (*open asterisk*; in **m**). **n** A detailed view of the framed area in **m**, showing the border between the ependyma (*arrowhead*) and the astrocyte layer (*asterisk*). The area in the fourth ventricle in **o** is shown in **p**. A robust layer of GFAP+ astrocytes lines the floor of the denuded ventricle. *Scale bars* **a**–**d** 150 μm, **e**–**l** 10 μm, **m** 300 μm, **n** 5 μm, **o** 20 μm, **p** 200 μm; *insets* in **a**–**d** 500 μm

In mature wt mice, the multiciliated ependyma formed a single cell layer that was readily recognized by the expression of the S100β protein (Fig. 1a, e), tubulin βIV (Fig. 1f) and the intermediate filament protein vimentin (Fig. 1h). The ciliated ependyma did not express GFAP (Fig. 1g).

In mature hyh mice, most of the ventricular surface lacked ependyma due to its disruption during development (Fig. 1b, d) [29, 41]. Nevertheless, multiciliated ependymal cells that resisted denudation remained in situ as small patches that were consistently located at specific sites of the ventricular walls (Fig. 1b, d) [29, 41, 65]. In the areas lacking ependyma, astrocytes formed a glial layer that covered the denuded ventricular surface. These cells lacked cilia (Fig. 1j) and expressed the intermediate filament proteins GFAP (Fig. 1k, o, p) and vimentin (Fig. 1l).

### Junction proteins in multiciliated ependyma and astrocytes lining the ventricular walls of wt and hyh mice

**Fig. 2:**
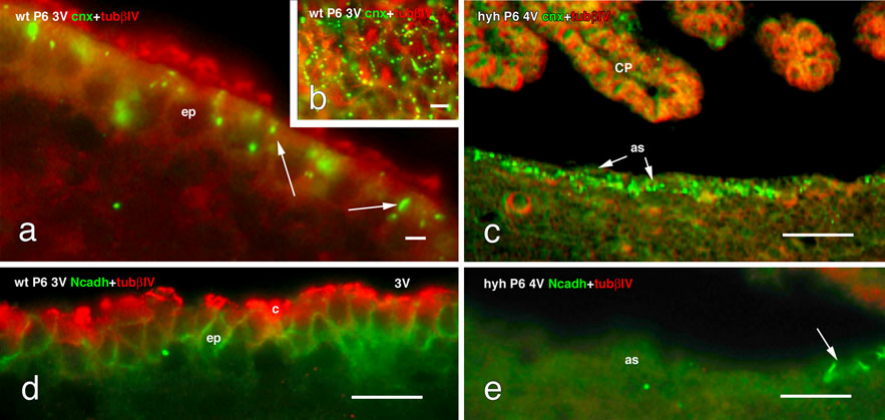
Expression of junction proteins in the cell lining the floor of the fourth ventricle of wt and hyh mice at P6. **a**–**c** Double immunofluorescence for tubulin βIV (*tubβIV,* *red*) and connexin 43 (*cnx*, *green*). **a** The multiciliated ependyma (*ep*) in wt mice present cnx+ spots localized preferentially at the apical cell pole of ependymal cells (*arrow*s). **b** Tangential section through the apical cell poles of the ependyma showing the distribution of cnx as *dots* in the lateral plasma membrane. **c** The astrocyte layer (*as*) lining the denuded ventricle in the hyh mouse expresses cnx (*arrow*) but not tubβIV. **d**, **e** Double immunofluorescence for tubβIV (*red*) and N-cadherin (*Ncadh*, *green*). **d** The ependyma in wt mice shows the belt-like distribution of Ncadh. **e** The astrocytes lining the denuded ventricles do not express Ncadh. *Arrow* points to a few ependymal cells remaining in situ and expressing Ncadh. *3V* third ventricle, *4V* fourth ventricle, *c* cilia, *CP* choroid plexus. *Scale bars* **a** 6 μm, **b** 8 μm, **c** 40 μm, **d** 12 μm, **e** 12 μm

The junction protein N-cadherin was present at the lateral plasma membrane forming a continuous belt around the apical cell pole of the multiciliated ependymal cells of wt and hyh mice (Fig. 2d). However, the layer of reactive astrocytes covering the denuded areas did not express N-cadherin (Fig. 2e).

The multiciliated ependymal cells of wt and hyh mice express connexin 43. This junction protein appeared as supranuclear granules and slender patches (possibly connexons) at the lateral plasma membrane (Fig. 2a, b). In the reactive astrocytes layer, connexin 43 appeared as granules throughout the cytoplasm (Fig. 2c). The older astrocyte layer of P30 mice apparently expressed more connexin 43 than did the P6 mice.

### Molecules implicated in transport mechanisms in multiciliated ependyma and astrocytes covering the ventricular walls of wt and hyh mice

**Fig. 3:**
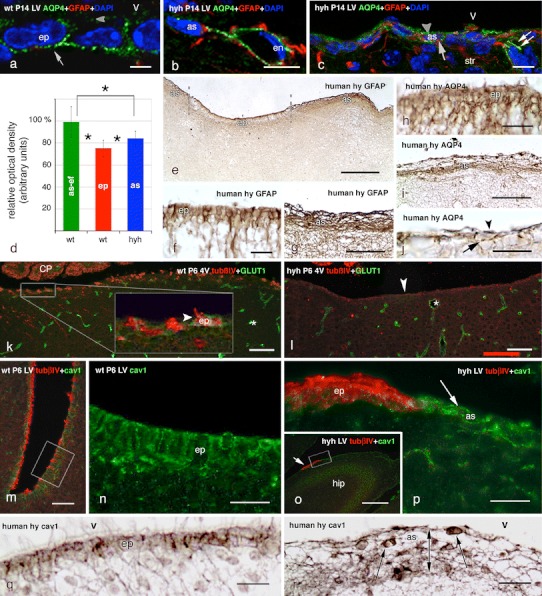
Expression of aquaporin 4, the glucose transporter 1 (GLUT1) and caveolin-1 in the ependyma of wt mice and in the astrocyte layer that covers the ependymal-denuded surface in hyh mice and a human hydrocephalic foetus. **a** Immunolabelling for aquaporin 4 (*AQP4*, *green*) in the ependyma (*ep*) of the lateral ventricle (*LV*) of a wt mouse; DAPI counterstaining (*blue*). The water channel is mainly located in the baso-lateral plasma membrane domain (*arrow*), but there is also a weak reaction at the apical domain (*arrowhead*). **b**, **c** Latero-medial wall of a hyh mouse with double immunofluorescence for GFAP (*red*), AQP4 (*green*) and DAPI counterstaining (*blue*). AQP4 immunoreaction is present in a perivascular astrocyte (*as*) and its endfeet surrounding endothelial (*en*) cells (**b**). Reactive astrocytes present AQP4 at the apical (*arrowhead*) and basal (*arrow*) cytoplasm and in their cell processes (*double arrow*) (**c**). **d** Optical density of the immunoreaction for AQP4 was recorded at (1) the ependyma (*ep*) of the latero-medial wall of the lateral ventricles of wt mice; (2) the cell layer of reactive astrocytes lining denuded areas of the latero-medial wall of the lateral ventricles of hyh mice (*as*); (3) the perivascular endfeet of the astrocytes (*as*-*ef*) of wt mice. Data represent the mean and standard deviation from four wt and four hyh mice (3–4 sections each mouse; four neighbour areas from each section). Data are expressed as relative percentage of the values obtained in each section where the mean of as-ef in wt mice was considered to be 100 %. *Correlation analysis showed significant differences (*p* < 0.001, Student’s *t* test). **e**–**j** Lateral ventricle of a 40-week-old human hydrocephalic foetus. Immunostaining for GFAP (**e**–**g**) and AQP4 (**h**–**j**). **e** Low power view showing ependyma not yet disrupted (*ep*) and denuded areas lined by a layer of GFAP+ astrocytes (*as*). These two regions are shown at higher magnification in **f** and **g**. The ependyma (**h**) and the astrocyte layer (**i**, **j**) express AQP4. **k**, **l** Fourth ventricle (*4V*) with double immunofluorescence for tubulin βIV (*tubβIV*, *red*) and GLUT1 (*green*). Endothelial cells (*asterisks*) are reactive to GLUT1. **k** The *inset* shows a weak reaction for GLUT1 in the ependyma lining the floor of the fourth ventricle of a P6 wt mouse. **l** The astrocyte layer lining the denuded floor of the fourth ventricle of hyh mice does not express GLUT1. **m** Lateral ventricle of a wt P6 mouse. Double immunofluorescence for tubβIV (*red*) and caveolin-1 (*cav1*, *green*). The framed area is shown in **n** using only the cav1 channel. **n** Detailed view of area framed in **m**, showing the strong expression of cav1 in the multiciliated ependyma of the lateral ventricle. **o** Wall of the lateral ventricle close to the hippocampus (*hip*) of a P6 hyh mouse. Double immunofluorescence for tubβIV (*red*) and cav1 (*green*). The walls are denuded with the exception of a tubβIV+ resistant ependymal patch (*arrow*). The area framed is shown in **p**. **p** Astrocytes lining the denuded areas of the lateral ventricle strongly express cav1 (*arrow*). The patch of ependyma is strongly labelled for tubβIV (*red*), whereas the astrocyte layer is not. **q**, **r** Lateral ventricle of a 40-week-old hydrocephalic human foetus. Section adjacent to that shown in **f** and **g**, immunostained for cav1. The cell body of ependymal cells contains immunoreactive granules (**q**). The cell body (*arrows*) and processes of the astrocytes forming the thick cell layer lining denuded areas (*double*-*ended* *arrow*) are immunoreactive (**r**). *CP* choroid plexus, *V* ventricle lumen. *Scale bars* **a** 5 μm, **b**, **c** 10 μm, **e** 200 μm, **f**, **h**, **j** 20 μm, **g** 50 μm, **i** 50 μm, **k**, **o** 100 μm, **l** 80 μm, **m** 40 μm, **n**, **p** 20 μm

In wt mice, the water channel protein aquaporin 4 was detected in the latero-basal cell domains, and less intensively in the apical domain, of the ependymal cells (Fig. 3a) and in the perivascular endfeet of the astrocytes. Aquaporin 4 was not detected in the cell bodies of the astrocytes. In contrast, in the hyh mouse, aquaporin 4 was found throughout the cell body and in the processes of the astrocytes covering the ependymal-denuded areas (Fig. 3c; Supplementary Fig. 3), as well as in the perivascular endfeet of the parenchymal astrocytes (Fig. 3b). Densitometric analysis showed that in hyh mice, the reactive astrocytes covering the ependyma-denuded surfaces presented a small but significant increase in immunoreactive aquaporin 4 with respect to the ependyma of wt mice (Fig. 3d).

The lateral ventricles of human hydrocephalic foetuses displayed large areas of ependymal denudation that clearly contrasted with those still lined by ciliated ependymal cells (cf. [18]; Fig. 3e–g). The denuded areas were covered by a layer of astrocyte cell bodies and processes (Fig. 3g). In the ependyma, aquaporin 4 outlined the cell profile (Fig. 3h). In the astrocytes, the water channel was present in the cell body and the processes (Fig. 3i, j).

The glucose transporter GLUT1 was weakly expressed in the multiciliated ependyma lining the fourth ventricle of P6 wt mice (Fig. 3k). It was not detectable in the ependyma of P30 wt mice or in the astrocytes covering the ependyma-denuded areas (Fig. 3l). In contrast, the immunoreaction was strong in the endothelial cells of wt and hyh mice (Fig. 3k, l).

The protein caveolin-1 labelled caveolae in a dotted pattern at the plasma membrane and cytoplasm of the multiciliated ependyma of mice (Fig. 3m, n) and of human foetuses (Fig. 3q). Caveolin-1 was detected throughout the cell body and the processes of astrocytes covering the ependyma-denuded areas in hyh mice (Fig. 3o, p) and in human hydrocephalic foetuses (Fig. 3r). This finding is in contrast with the poor or absent immunoreaction found in parenchymal astrocytes.

### Endocytosis in multiciliated ependyma and in the astrocyte layer lining the ependyma-denuded regions of hyh mice

**Fig. 4:**
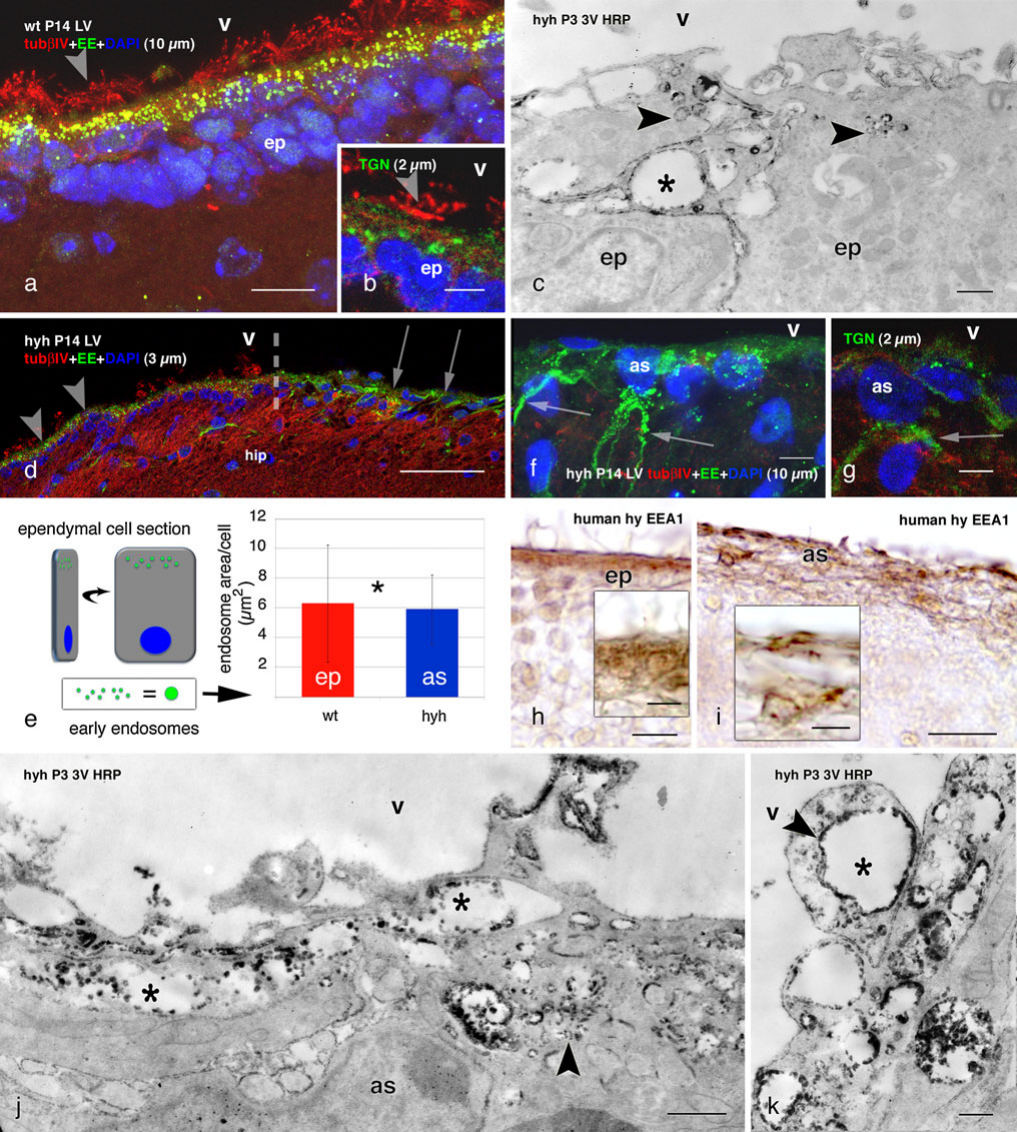
Endocytosis of HRP injected in vivo into the lateral ventricle of wt and hyh mice, and location of endocytic vesicles, early endosomes and the Golgi apparatus. Lateral ventricle of wt (**a**, **b**) and hyh (**d**, **f**, **g**) mice, at P14. Confocal laser microscopy of immunolabelling for the EEA1 antigen of early endosomes (*EE*, *green* in **a**, **d**, **f**), the trans-Golgi network (*TGN*, *green* in **b**, **g**) and tubulin βIV (*tubβIV*, *red*). DAPI nuclear staining (*blue*). Z-projections comprising confocal planes for different thicknesses (*between brackets*). Numerous EE are present in the apical pole (*arrowheads* in **a**, **d**) of the ependymal cells (*ep*) in wt and hyh mice. The *broken line* in **d** shows the border between a patch of intact ependyma (*arrowheads*) and the astrocyte layer covering an ependyma-denuded surface (*arrows*) in the hippocampus (*hip*). The TGN in ependymal cells is located juxtanuclear (*arrowhead* in **b**). In the astrocytes (*as*) covering the ependyma-denuded surface of hyh mice (*arrows* in **d**), abundant EE and TGN are present in the cell bodies and processes (*arrows* in **f** and **g**). **e** Total area occupied by EEA1-reactive EE per ependymal cell of wt mice and per reactive astrocyte (*as*) of hyh mice in confocal laser cuts of frozen sections (explanation on the *left side of the figure*). Data represent the mean and standard deviation obtained from sections corresponding to four wt and four hyh mice (4 sections each mouse). *Correlation analysis did not show a significant difference (*p* = 0.695, Student’s *t* test). **c**, **j**, **k** Ultrastructural detection in the third ventricle (*3V*) wall of HRP injected into a lateral ventricle at P3 in wt mice (**c**) and hyh mice (**h**, **i**). In the apical pole of ependymal cells and in astrocytes, HRP is located within endocytic vesicles (*arrowheads*) and large EE (*asterisks*). **h**, **i** Lateral ventricle of a 40-week-old human hydrocephalic foetus. Section adjacent to that shown in Fig. 3f, g, immunostained for EEA1. The cell body of ependymal cells (*ep*) contains immunoreactive granules (**h**). The cell body and processes of the astrocytes forming the thick cells layer lining denuded areas are immunoreactive (**i**). *LV* lateral ventricle, *str* striatum, *V* ventricle lumen. *Scale bars* **a** 10 μm, **b**, **g** 5 μm, **c**, **j** 400 nm, **d** 50 μm, **f** 7 μm, **h**, **i** 20 μm; *Insets* in **h**, **i** 10 μm, **k** 200 nm

Early endosomes, immunodetected by the presence of the EEA1 antigen, were present in the apical juxtanuclear domain of the multiciliated ependymal cells of wt and hyh mice (Fig. 4a, d). The trans-Golgi network was detected in the supranuclear region below the layer of the early endosomes (Fig. 4b). HRP administered in vivo into the lateral ventricle of wt and hyh mice was traced with the electron microscope. In both the normal ependyma of wt mice and the denudation-resistant ependyma of hyh mice, HRP was visualized within small endocytic vesicles and in large irregular compartments that corresponded to early endosomes (Fig. 4c; Supplementary Fig. 4).

At variance with the parenchymal astrocytes, the astrocytes covering the ependyma-denuded areas of hyh mice displayed numerous EEA1-positive endosomes scattered throughout the cell body and processes (Fig. 4d, f). In these astrocytes, the cytoplasm area occupied by the early endosomes was not significantly different from that of the ependymal cells of wt mice (Fig. 4e); the trans-Golgi network was located in a cytoplasmic region that was close to the ventricle (Supplementary Fig. 5) and along the processes (Fig. 4g). HRP administered in vivo to hyh mice was incorporated into small endocytic vesicles and early endosomes of astrocytes covering denuded areas (Fig. 4j, k; Supplementary Fig. 4).

The ependyma of the lateral ventricles of human hydrocephalic foetuses displayed a strong EEA1 immunoreactivity in the supranuclear cytoplasm (Fig. 4h). The cell body and processes of the astrocytes lining the adjacent denuded areas were strongly reactive with anti-EEA1 (Fig. 4i).

### Paracellular routes of transport in ependyma of wt mice and astrocytes covering ependyma-denuded areas in hyh mice

**Fig. 5:**
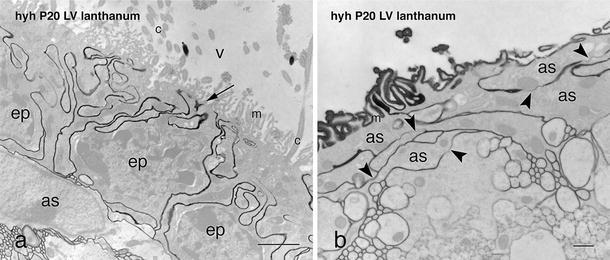
Ultrastructural detection of lanthanum nitrate applied into the lateral ventricle of a P20 hyh mouse. **a** Lanthanum penetrates from the ventricular lumen (*V*, *arrow*) towards the brain parenchyma through the winding extracellular spaces of the denudation-resistant, ciliated ependyma (*ep*). **b** In the astrocytic layer (*as*) lining the denuded ventricular surface of a hyh mouse, the tracer penetrates through the extracellular spaces and bypasses the gap junctions joining the astrocytes (*arrowheads*). *c* cilia, *LV* lateral ventricle, *m* microvilli. *Scale bars* **a** 2 μm, **b** 500 nm

On electron microscope, the lateral plasma membranes of neighbouring ependymal cells were found to be extensively interdigitated (Fig. 5a; Supplementary Fig. 4) and joined together by adherents and gap junctions; tight junctions were missing. Lanthanum applied to the ventricular surface penetrated through the labyrinth of extracellular spaces, filled the intercellular space of the underlying neuropile and labelled the basement membrane of the capillaries and the intercellular space of the endothelium up to the tight junctions joining the endothelial cells (Supplementary Fig. 5). Lanthanum tracing further supported the absence of tight junctions at the multiciliated ependymal lining.

Electron microscope analysis revealed that astrocytes projected into the ventricle numerous, irregularly shaped, microvilli; cilia were missing (Figs. 1m, n, 5b). These cells displayed numerous sheet-like processes that interdigitated extensively with those of adjacent cells, forming a dense subventricular network of astrocyte processes (Fig. 5b; see Supplementary Fig. 5) that was readily visualized with GFAP immunocytochemistry (Fig. 1k, p). The surface astrocytes were joined together by gap junctions (Fig. 5b) and lacked zonula adherens and tight junctions (Fig. 5b; Supplementary Figs. 4 and 5).

**Fig. 6:**
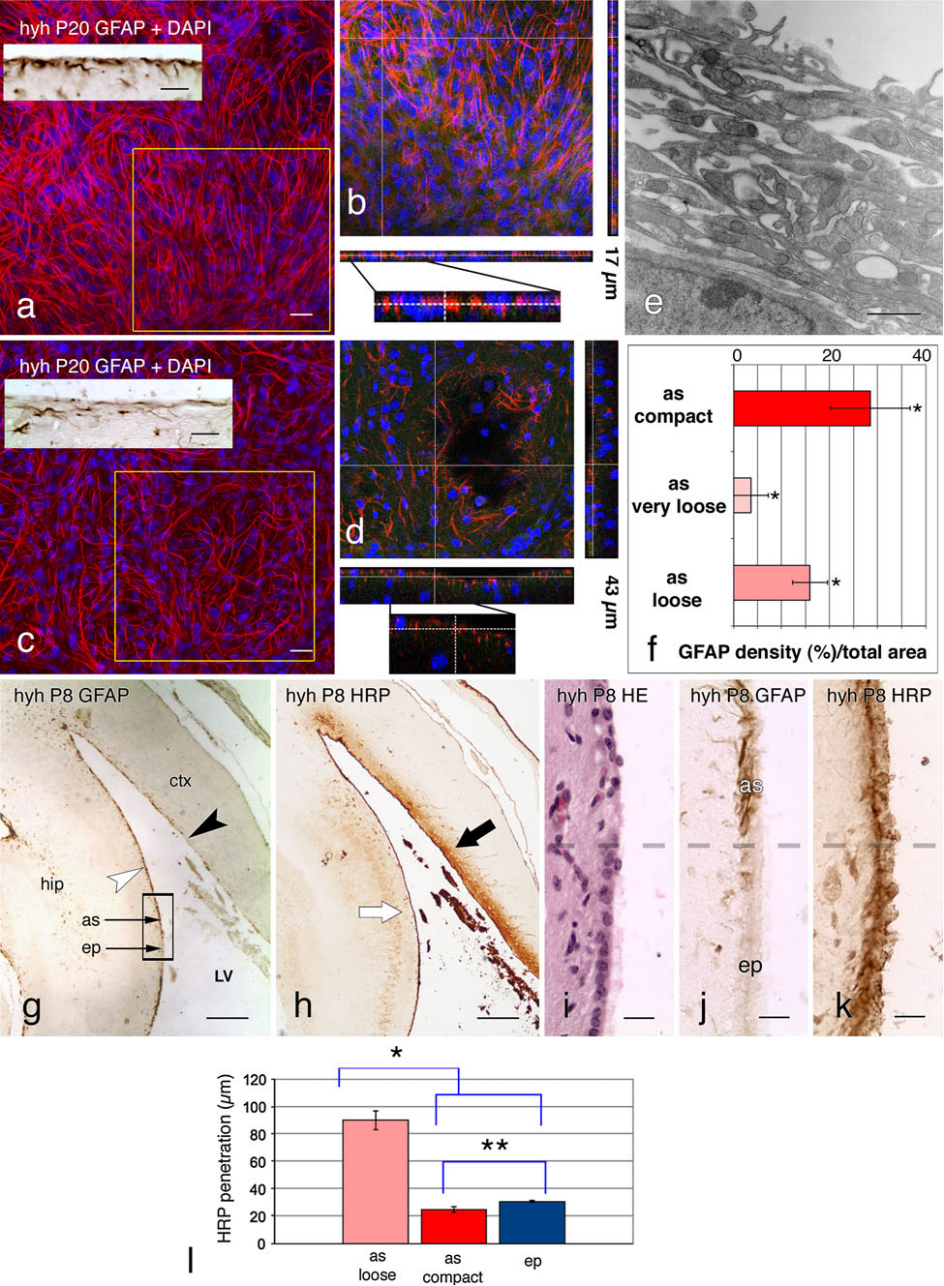
Tightness and permeability of the layer of astrocytes lining different denuded regions of the lateral ventricle of hyh mice. **a**–**d** Hyh mice at P20. Surface views obtained from whole mount preparations of ventricular walls processed for GFAP (*red*) immunofluorescence and DAPI nuclear staining. The astrocyte cell density in the latero-medial wall of the lateral ventricle (**a**, **b**) is much higher than that of the latero-external wall of the lateral ventricle (**c**, **d**; for orientation, see **g**). **b**, **d** 1 μm confocal planes of the areas framed in **a** and **b** displaying pseudo 3D reconstructions (17 μm in **b**, 43 μm in **d**) bring out the differential arrangement of the astrocytes. *Insets* in **a**, **c** immunocytochemistry for GFAP in sections of similar areas. **e** Ultrastructure of the latero-external wall of a lateral ventricle of a hyh mouse at P20 showing the loose organization of astrocytes. **f** Measurement of astrocyte (*as*) cell densities using whole mount preparations of regions of the lateral ventricle displaying compact (latero-medial wall, see **a**), loose (see **c**) or very loose (latero-external walls) arrangements of astrocytes. Data represent mean and standard deviation of the percentage of the area occupied by the GFAP-immunoreactive profiles with respect to the total area. Data were collected from four hyh mice, four with whole mounts/mouse/each location. *Correlation analysis showed significant differences between the three types of astrocyte organization (*p* < 0.001, Student’s *t* test). **g** Frontal section through the telencephalon of a hyh mouse at P8 immunostained for GFAP. The latero-medial wall of the lateral ventricle is covered by an astrocytic layer (*as*; *white arrowhead*) and a few patches of ependyma (*ep*). The area framed is shown in **i**–**k**. The latero-external wall of the lateral ventricle contains a discontinuous astrocytic layer and scattered ependymal cells (*black arrowhead*). **h** Adjacent section to that shown in **g**, immunostained using anti-HRP to visualize the HRP injected in vivo into a lateral ventricle. In the latero-medial wall of the ventricle, the tracer is incorporated equally by the layer of astrocytes and the patch of ependyma (*white arrow*). In the latero-external wall of the ventricle, HRP penetrates deeply into the brain parenchyma (*black arrow*). **i**–**k** Adjacent serial sections through an area similar to that framed in **g**, including a patch of ependyma lying close to a denuded area lined by an astrocytic layer. The *broken line* points to the border between both areas. Haematoxylin–eosin staining (**i**), anti-GFAP (**j**) and anti-HRP (**k**) immunolabelling. The astrocyte layer and the ependymal patch incorporate HRP following a similar pattern. **l** Measurement of the penetration of intraventricularly injected HRP into the brain parenchyma at three different regions of the lateral ventricles: with ependyma (**j**, **k**), compact layer of reactive astrocyte (*white arrow*/*arrowhead* in **g**, **h**) and loose layer of astrocytes (*black arrow*/*arrowhead* in **g**, **h**). Data represent mean and standard deviations from four hyh P8 mice (4 measurements in 3–4 sections from each mouse). *The correlation analysis showed a significant difference between the degree of penetration of HRP through the loose astrocyte layer and that of the other two regions (*p* < 0.001, Student’s *t* test). **There was not a significant difference between the data from the penetration through the astrocyte compact organization and the ependyma in the hippocampus of hyh mice (*p* = 1.119, Student’s *t* test). *ctx* cerebral cortex, *hip* hippocampus, *LV* lateral ventricle. *Scale bars* **a**, **c** *insets* in **a** and **c** 20 μm, **e** 1 μm, **g**, **h** 200 μm, **i**–**k** 20 μm

In hyh mice, the astrocyte layer covering the denuded brain ventricles showed regional differences. The astrocytes formed a cell layer with varying degrees of tightness that ranged from loose to compact. In the latero-medial wall of the lateral ventricles lining the hippocampus and striatum (Figs. 1k, 6a, b, g), the third ventricle, the ventral wall of the cerebral aqueduct and the fourth ventricle (Fig. 1o, p), the denuded surface was lined by densely packed astrocytes. In other denuded areas, such as those of the dorsal and external walls of the lateral ventricles, reactive astrocytes were arranged as a loose cell layer (Fig. 6c–e). The different degrees of cell density of the astrocyte assembled at the denuded areas became most evident in whole mount preparations of different ventricular regions immunostained for GFAP (Fig. 6a–d) and used for a densitometric analysis (Fig. 6f). This study led to identify three types of astrocyte arrangements: (1) compact, characterized by a continuous layer of tightly packed astrocytes (Figs. 1o, p, 2c, 4b, 6a, b); (2) loose, recognized by a continuous layer of astrocytes separated by wide intercellular spaces (Fig. 6c–e); (3) very loose, characterized by a discontinuous layer of astrocytes (Fig. 6c, inset).

The barrier property, in terms of paracellular permeability, for these different types of astrocyte arrangements located at distinct regions of the ventricular system was tested by in vivo administration of HRP. Five minutes after HRP injection into a lateral ventricle of hyh mice, the tracer was incorporated by the dense astrocytic layer and penetrated about 20 μm into the underlying neuropile, resembling the barrier property of the neighbouring patch of multiciliated ependyma (Fig. 6h–l). In the areas that displayed loosely arranged astrocytes, HRP penetrated about 90 μm into the brain parenchyma (Fig. 6g, h), indicating a rather free movement of HRP at this level.

## Discussion

In hyh hydrocephalic mice, there is a programme of neuroepithelium/ependyma denudation starting early in foetal life and ending by the end of the first postnatal week; the missing ependyma is replaced by a layer of astrocytes forming a new interface between the CSF and the brain parenchyma [29, 41, 65]. This phenomenon has also been described in human hydrocephalic foetuses [18, 53, 55]. The present study, carried out in hyh mice and human cases, has revealed that the new surface layer of astrocytes shares some phenotypic and functional features with the ependyma [summarized in Table 3], suggesting that such a unique astrocyte reaction may represent an attempt to re-establish some lost functions at the brain parenchyma–CSF interface.

**Table 3:** Summary of results displaying the presence (+) or absence (−) of the structural and functional markers in the hyh mouse

	Cytoskeleton	Gap junctions (connexin 43)	Adherens junctions (N-cadherin)	Microvilli	Glucose transport (GLUT1)	Water transport (aquaporin 4)	Endocytosis/transcytosis (caveolae containing caveolin 1)	Endocytosis/transcytosis (endosomes containing EEA1 antigen	Endocytosis/transcytosis (HRP uptake)	Absence of tight junctions/paracellular permeability (lanthanum tracing)
Ependyma	(GFAP)^a^ Vimentin	+	+	+	±^b^	**+**	**+**	**+**	+	+
Cell layer of reactive astrocytes	GFAP, vimentin	+	–	+	–	**+**	**+**	**+**	+	+

Findings in bold have been also obtained in human foetuses

^a^At variance with mouse ependyma, the ependyma of human foetus is reactive with anti-GFAP

^b^GLUT1 is only present in immature ependymal cells

### The astrocytes covering the denuded ventricular walls of hydrocephalic hyh mice form a new cell layer with a cell organization that resembles the ependyma

**Fig. 7:**
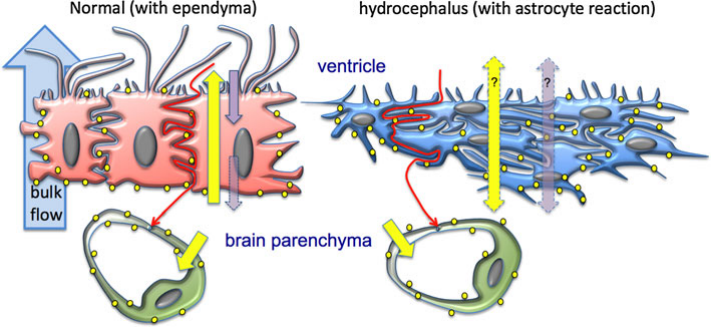
Schematic representation of the transcellular and paracellular transport mechanisms that would operate at the ependyma and the layer of reactive astrocytes. In the ependymal cells of wt mice (*left*), most of the aquaporin 4 (*yellow dots*) is located at the basolateral domains (some is found at the apical domain), suggesting that the ependyma transports water from the brain parenchyma (*bottom*) towards the ventricular CSF (*upper*) (*yellow arrow* across the ependyma). There are pinocytic processes and transcytosis directed in the opposite direction through this barrier (*purple arrow*). In hyh mouse (*right*), a layer of reactive astrocytes cover the ependyma-denuded surfaces. These cells express aquaporin 4 throughout the cell body and processes (*yellow dots*) and could be involved in water transport from or to the CSF (*double*-*headed yellow arrow*). The pinocytic processes in the astrocytes could also operate in both directions (*double head purple arrows*). In addition, as in the normal situation, parenchymal astrocytes together with the endothelial cells would be involved in an aquaporin 4-mediated transport towards the brain capillaries. Both barriers would transport molecules from the CSF to the brain parenchyma through a paracellular route (*winding red arrows*)

The astrocytes found at the denuded ventricular walls form a new cell layer that in several aspects resembles the ependyma; i.e. it expresses vimentin, lacks tight junctions, displays connexin 43-based gap junctions, projects numerous microvilli to the ventricle and displays numerous lateral interdigitations that result in a winding intercellular space (Fig. 7). The existence of gap junctions between ependymal cells has been widely demonstrated [6, 8, 9, 21, 28], and they are believed to play a role in the synchronization of cilia beating [50, 55]. Their functional significance in the astrocyte layer is unknown; they could be associated with electrical and metabolic activities, the determination of cell phenotype [45], or the clearance of cytotoxic molecules and the spreading of neuroprotective factors that takes place in brain injuries, ischaemia and hypoxia [27, 33, 37, 51, 58, 60]. Whatever the functional significance of the gap junctions connecting the astrocytes covering the denuded ventricular surface, it may be suggested that these cells are coupled to play a function as a CSF–brain barrier involved in water and solute transport (Fig. 7).

### The ependyma and the astrocyte cell layer present active endocytic mechanisms

Ependymal cells incorporate CSF proteins into the pinocytic-lysosome pathway [14]. The multiciliated ependyma of wt mice and the denudation-resistant ependyma of hyh mice express caveolin-1, which is in agreement with the expression of EEA1 antigen, a reliable marker of early endosomes, in these cells (present report). Early endosomes are dynamic cell compartments that are involved in endocytosis and sorting mechanisms [32, 67]. Caveolin-1 is a protein that is present in caveolae, structures that play a role in endocytosis and transcytosis [20, 23]; caveolin is also present in both the ependyma [44, 50, present report] and the reactive astrocytes [present study]. Although most cargo that is endocytosed via caveolae is fluid, certain compounds enter into caveolae via specific receptors [62]. The presence of caveolae in the ependyma is in agreement with the known capacity of ependymal cells to incorporate tracer molecules present in the CSF [8, 10]. In the reactive astrocytes, caveolae can play a similar role, which is in agreement with the existence of endocytosis and sorting mechanisms [32, 67], which have also been experimentally tested in the present study by injecting HRP into the CSF of living mice. At variance with the ependyma, the non-polarized distribution of caveolae and endosomes in the reactive astrocytes indicate that they can incorporate fluid and substances not only from the ventricle but also from the parenchymal fluid (Fig. 7).

### Role of the expression of the water channel protein aquaporin 4 in the ependyma and in the new astrocyte cell barrier

Aquaporin 4 is a water channel with marked prevalence in periventricular areas [63]. In the ependymal cells, it is located in the latero-basal domains [46, present report] and, less extensively, in the apical domain (present report). In parenchymal astrocytes, it is mostly found in the vascular endfeet [46, present report]. At variance, reactive astrocytes lining the denuded ependyma in hydrocephalic mice and human foetuses overexpress aquaporin 4, and this protein is found throughout the cell body and processes (Fig. 7). The presence of aquaporin 4 in parenchymal reactive astrocytes has been proposed to be involved in the water entry to astrocytes in initial stages of oedema formation [38] to re-establish the osmotic equilibrium [64]. The periventricular reaction found in the present report, both in mice and humans, could represent an attempt to re-establish the equilibrium between the ventricular and parenchymal fluids, or to help CSF transport from ventricles to brain capillaries (Fig. 7). Ependymal aquaporin 4 has been proposed to play a protective role in hydrocephalus by allowing for the transependymal re-absorption of CSF into brain capillaries [56]. The presence of aquaporin 4 in the apical plasma membrane of ependymal cells supports this possibility. It appears that aquaporin 4 may have a relevant role in hydrocephalus and be a useful therapeutic target [34, 54; reviewed by 40, 42, 56].

### The astrocyte layer and the ependyma present similar paracellular routes of transport

The multiciliated ependyma is an epithelial-like layer interposed between the CSF and the brain parenchyma. Although it does not behave like a tight barrier [8, 9], it seems to regulate the transport of ions, small molecules and water [12, 66]. Tight junctions are absent or poorly developed in the mature multiciliated ependyma [8, 12]. Lanthanum nitrate injected into the ventricle of wt and hyh mice further proves that the extracellular spaces of both, the ependyma and astrocyte cell layer, are not sealed (Fig. 7). Furthermore, the ependymal and glial barriers responded similarly to the in vivo intraventricular injection of HRP; the tracer moved through the intercellular space of both barriers and penetrated only a few micrometres into the underlying neuropile, suggesting that both barriers somehow limited the amount of HRP moving from the CSF to the brain parenchyma. This possibility is supported by the finding that HRP penetrated deeply into the brain parenchyma in the regions of the ventricular walls with a poorly developed astrocyte cell layer.

Different and distinct regions of the denuded ventricular walls trigger different astrocyte reactions that lead to glial layers with different degrees of cell density and tightness. What are the signals mediating these different responses? In hyh mice, ependymal denudation takes place at prenatal stages prior to detectable hydrocephalus [30]. Therefore, intraventricular pressure or expanding ventricles cannot be considered to be responsible for the absence of ependyma in these mice. Similarly, the periventricular astrocyte reaction appears shortly after denudation, when astrogliogenesis takes place but at a stage when ventriculomegaly is just starting to develop. Furthermore, the most robust astrocyte layer is that lining the denuded floor of the fourth ventricle, a cavity displaying no dilatation. The mechanism underlying the formation of the astrocyte layer replacing the lost ependyma is not known. Is the actual absence of the ependyma, or the direct exposure of the neuropile to the CSF, or a mechanical effect of the expanding ventricles involved? Are there different physiopathological consequences for brain regions protected by a new and compact layer of astrocytes and those close to a ventricular wall lined by a loose and highly permeable layer of astrocytes? These are open questions for future investigations.

## Electronic supplementary material

Below is the link to the electronic supplementary material.

Supplementary Figure 1. Hyh mouse at P6, beginning to develop a severe hydrocephalus. Frontal paraffin sections stained with haematoxylin-eosin at rostro-caudal levels from **a** to **f**. *Abbreviations*: *3V*, third ventricle, *4V*, fourth ventricle; *CA*, cerebral aqueduct; *VL*, lateral ventricle. *Scale bars*: **a**-**f**, 500 µm (TIFF 18455 kb)

Supplementary Figure 2. Schematic representation of the ventricles of a hyh mouse at P14 with full severe hydrocephalus. Frontal (**a**) and sagittal (**b**) views. *Black thick* lines show the ependyma resisting denudation. *Abbreviations*: *3V,* third ventricle; *4V*, fourth ventricle; *CA*, cerebral aqueduct; *Ceb*, cerebellum; *DREA*, denudation resistant ependyma of the aqueduct (see reference [19]); *Hb*, habenula; *HRc*, habenular recess; *PRc*, pineal recess; *ME*, median eminence (TIFF 13881 kb)

Supplementary Figure 3. Expression of aquaporin 4 and the EEA1 antigen in the astrocyte layer covering the cerebral aqueduct and the fourth ventricle of hyh mice, at P6 and P20. (**a, b**) Immunolabelling for aquaporin 4 in the cerebral aqueduct (**a**) and fourth ventricle (**b**) of a hyh mouse at P6. Brain capillaries (*arrows*) are labelled in addition to the reactive astrocytes. (**c, d**) Immunolabelling for the early endosomal antigen EEA1 (*green*) and GFAP (*red*) in the reactive astrocytes covering the cerebral aqueduct (**c**) and fourth ventricle (**d**) of a hyh mouse at P20. DAPI nuclear staining (*blue*). *Abbreviations*: *4V*, fourth ventricle; *AQP4*, aquaporin 4; *CA*, cerebral aqueduct; *CP*, choroid plexus; *EE*, early endosomes. *Scale bars*: **a**, **b**, 50 µm; **c**, **d**, 10 µm (TIFF 26806 kb)

Supplementary Figure 4. Fourth ventricle of wt and hyh mice at P20 and P30 after in vivo administration of HRP into a lateral ventricle and in vitro tracing with lanthanum nitrate. (**a**) Wt mouse**.** Lanthanum applied into the ventricle passed through the thin interwoven extracellular spaces of the ependyma lining the floor of the fourth ventricle (*arrowheads*). **(b)** Wt mouse**.** HRP is incorporated into early endosomes located at the apical pole of the ependyma (*large arrow*) and into the intercellular space (*small arrows*). **(c)** The denuded floor of the fourth ventricle of hyh mice is covered by a layer of densely packed reactive astrocytes. (**d**) Hyh mouse. Lanthanum applied into the ventricle passed through the thin interwoven extracellular spaces of the astrocyte layer (*arrow*). *Abbreviations*: *CP*, choroid plexus. *Scale bars*: **a**, 1 µm; **b**, **d**, 2 µm; **c**, 30 µm (TIFF 8258 kb)

**Supplementary Figure 5. Ultrastructural detection of lanthanum nitrate applied into the lateral ventricle of a hyh mouse, at P20**. (**a-c**; **c** is a detailed view of the area framed in **b**) Lanthanum penetrates from the ventricular lumen towards the brain parenchyma through the winding extracellular spaces of the astrocytic layer (*as*) lining the denuded ventricular surface (*black arrows*). Lanthanum reached the intercellular space of the neuropile and the pericapillary basement membrane, a transport pathway similar to that of the areas lined by ependyma. Tight junctions present in the endothelial cells (*en*; in **b**) prevent the extracellular progression of the tracer (*white arrows*; in **b**, **c**). (**d**) Tight junctions present in the choroid plexus ependyma also prevent the extracellular progression of the tracer (*white arrow*). *Asterisk*: intermediate filament bundles of astrocytes. *Abbreviations*: *CP*, Choroid plexus; *g*, Golgi apparatus dictiosomes; *LV*, lateral ventricle; *V*, intercellular space open to the ventricular lumen. *Scale bars*: **a**, 500 nm; **b**, 1 µm; **b**, 30 µm; **d**, 200 nm (TIFF 13478 kb)

Supplementary material 6 (DOCX 116 kb)
