# Supplementary figures and images for: Astrocytes acquire morphological and functional characteristics of ependymal cells following disruption of ependyma in hydrocephalus
Source: Acta Neuropathol. 2012 May 11;124(4):531–46. doi: 10.1007/s00401-012-0992-6 (PMC3444707; doi:10.1007/s00401-012-0992-6)

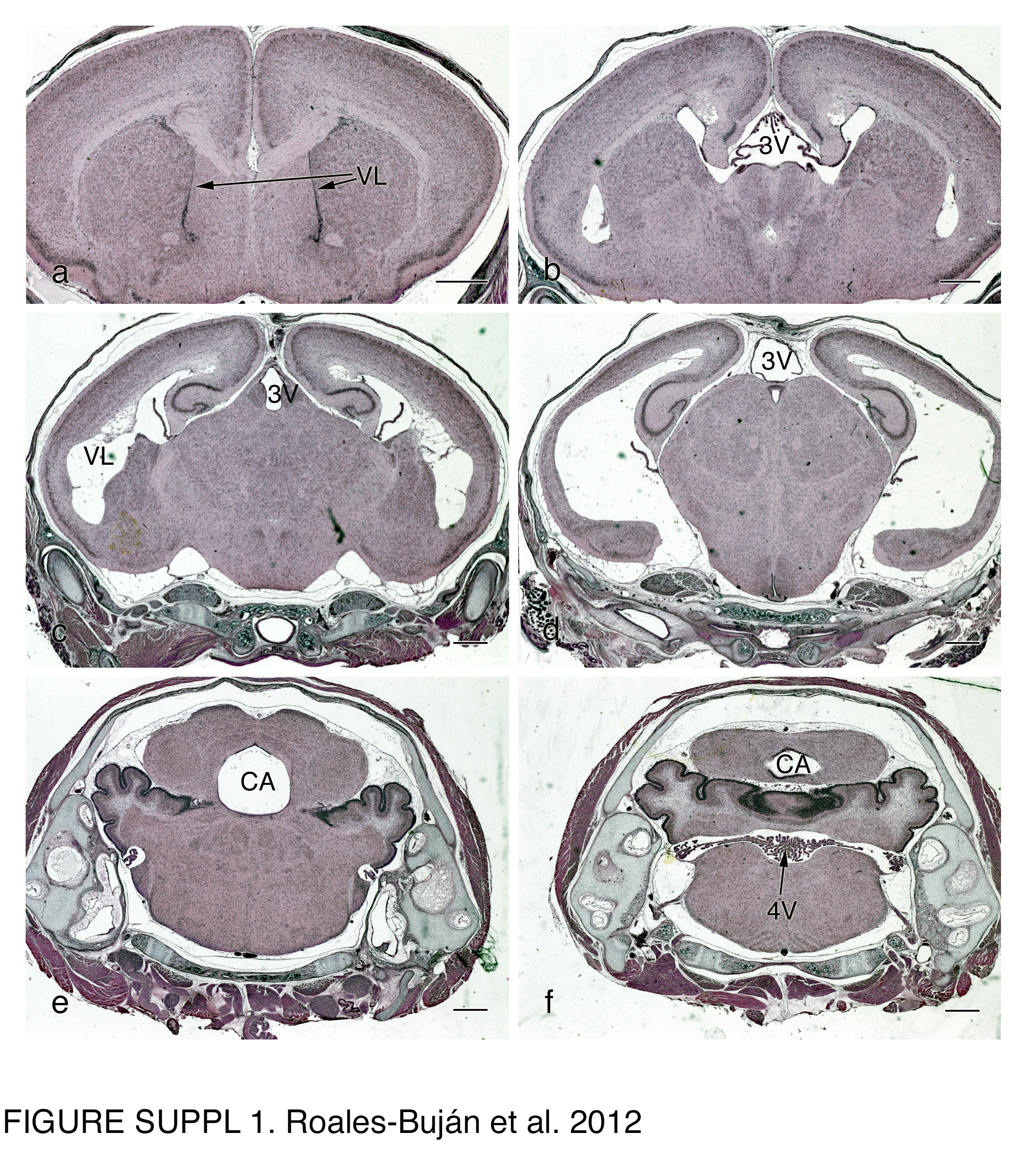

Supplement: Supplementary file 1 — Supplementary Figure 1. Hyh mouse at P6, beginning to develop a severe hydrocephalus. Frontal paraffin sections stained with haematoxylin-eosin at rostro-caudal levels from a to f. Abbreviations: 3V, third ventricle, 4V, fourth ventricle; CA, cerebral aqueduct; VL, lateral ventricle. Scale bars: a-f, 500 µm (TIFF 18455 kb) [file 401_2012_992_MOESM1_ESM.tif]

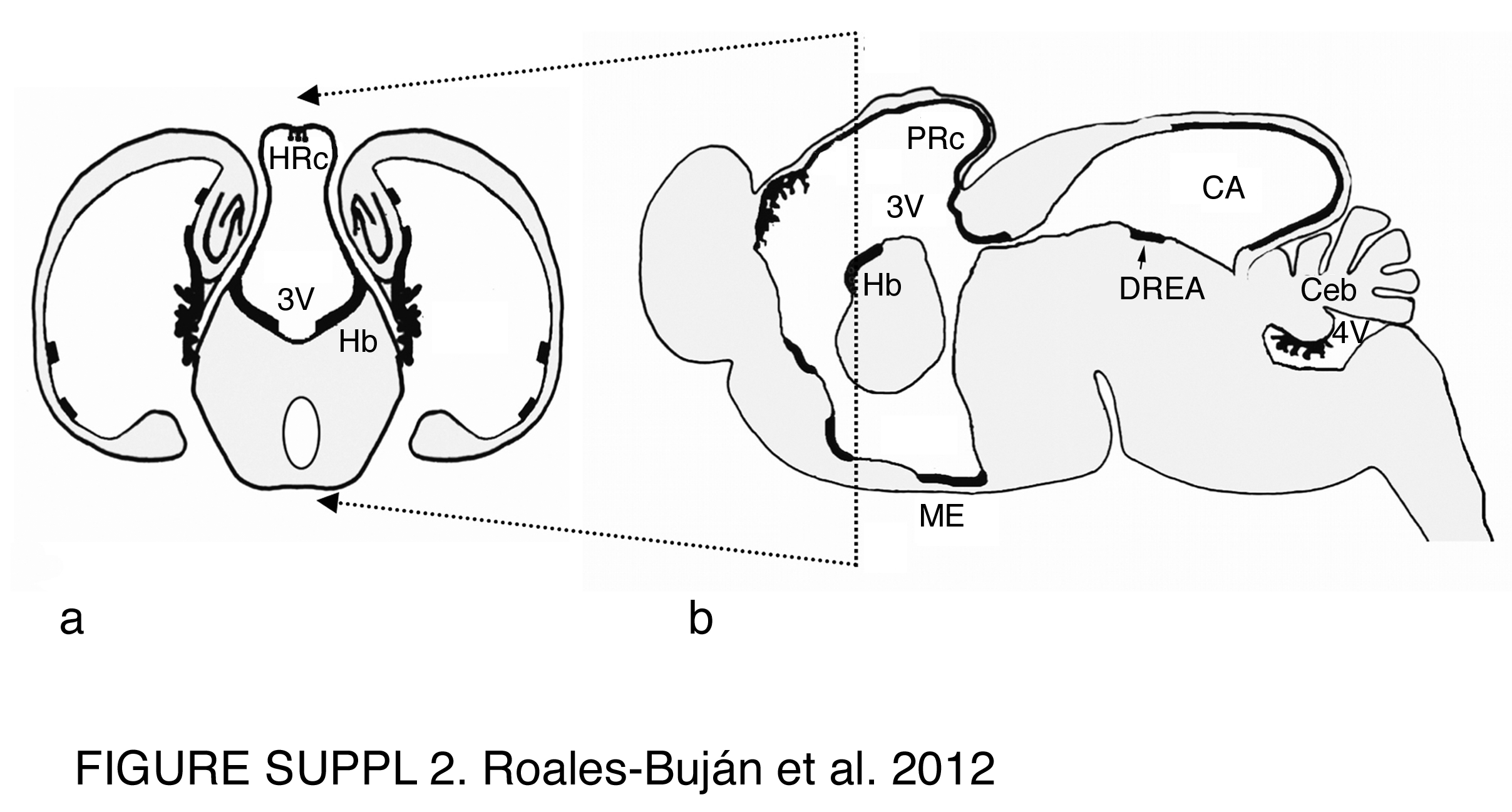

Supplement: Supplementary file 2 — Supplementary Figure 2. Schematic representation of the ventricles of a hyh mouse at P14 with full severe hydrocephalus. Frontal (a) and sagittal (b) views. Black thick lines show the ependyma resisting denudation. Abbreviations: 3V, third ventricle; 4V, fourth ventricle; CA, cerebral aqueduct; Ceb, cerebellum; DREA, denudation resistant ependyma of the aqueduct (see reference [19]); Hb, habenula; HRc, habenular recess; PRc, pineal recess; ME, median eminence (TIFF 13881 kb) [file 401_2012_992_MOESM2_ESM.tiff]

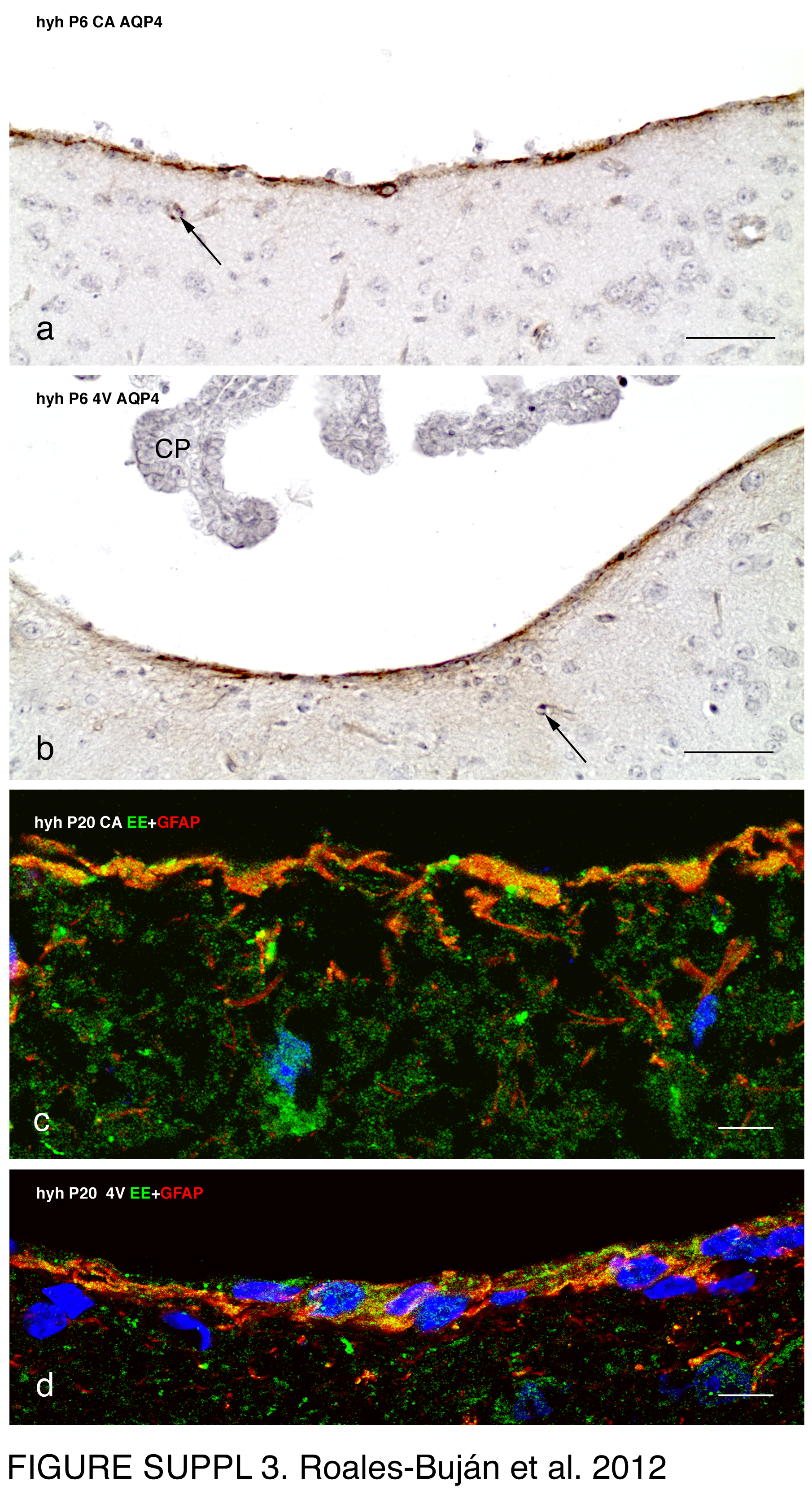

Supplement: Supplementary file 3 — Supplementary Figure 3. Expression of aquaporin 4 and the EEA1 antigen in the astrocyte layer covering the cerebral aqueduct and the fourth ventricle of hyh mice, at P6 and P20. (a, b) Immunolabelling for aquaporin 4 in the cerebral aqueduct (a) and fourth ventricle (b) of a hyh mouse at P6. Brain capillaries (arrows) are labelled in addition to the reactive astrocytes. (c, d) Immunolabelling for the early endosomal antigen EEA1 (green) and GFAP (red) in the reactive astrocytes covering the cerebral aqueduct (c) and fourth ventricle (d) of a hyh mouse at P20. DAPI nuclear staining (blue). Abbreviations: 4V, fourth ventricle; AQP4, aquaporin 4; CA, cerebral aqueduct; CP, choroid plexus; EE, early endosomes. Scale bars: a, b, 50 µm; c, d, 10 µm (TIFF 26806 kb) [file 401_2012_992_MOESM3_ESM.tif]

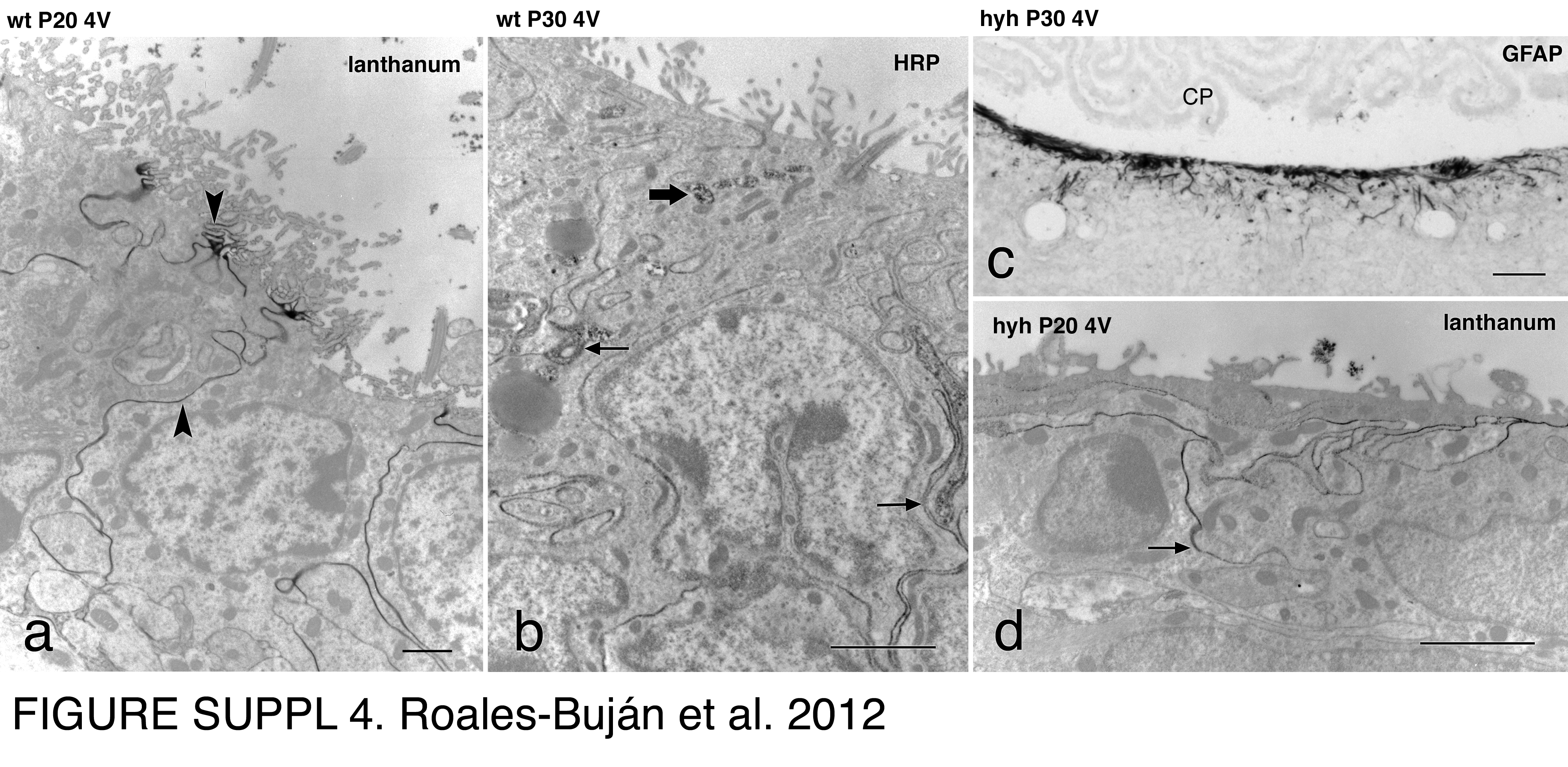

Supplement: Supplementary file 4 — Supplementary Figure 4. Fourth ventricle of wt and hyh mice at P20 and P30 after in vivo administration of HRP into a lateral ventricle and in vitro tracing with lanthanum nitrate. (a) Wt mouse. Lanthanum applied into the ventricle passed through the thin interwoven extracellular spaces of the ependyma lining the floor of the fourth ventricle (arrowheads). (b) Wt mouse. HRP is incorporated into early endosomes located at the apical pole of the ependyma (large arrow) and into the intercellular space (small arrows). (c) The denuded floor of the fourth ventricle of hyh mice is covered by a layer of densely packed reactive astrocytes. (d) Hyh mouse. Lanthanum applied into the ventricle passed through the thin interwoven extracellular spaces of the astrocyte layer (arrow). Abbreviations: CP, choroid plexus. Scale bars: a, 1 µm; b, d, 2 µm; c, 30 µm (TIFF 8258 kb) [file 401_2012_992_MOESM4_ESM.tiff]

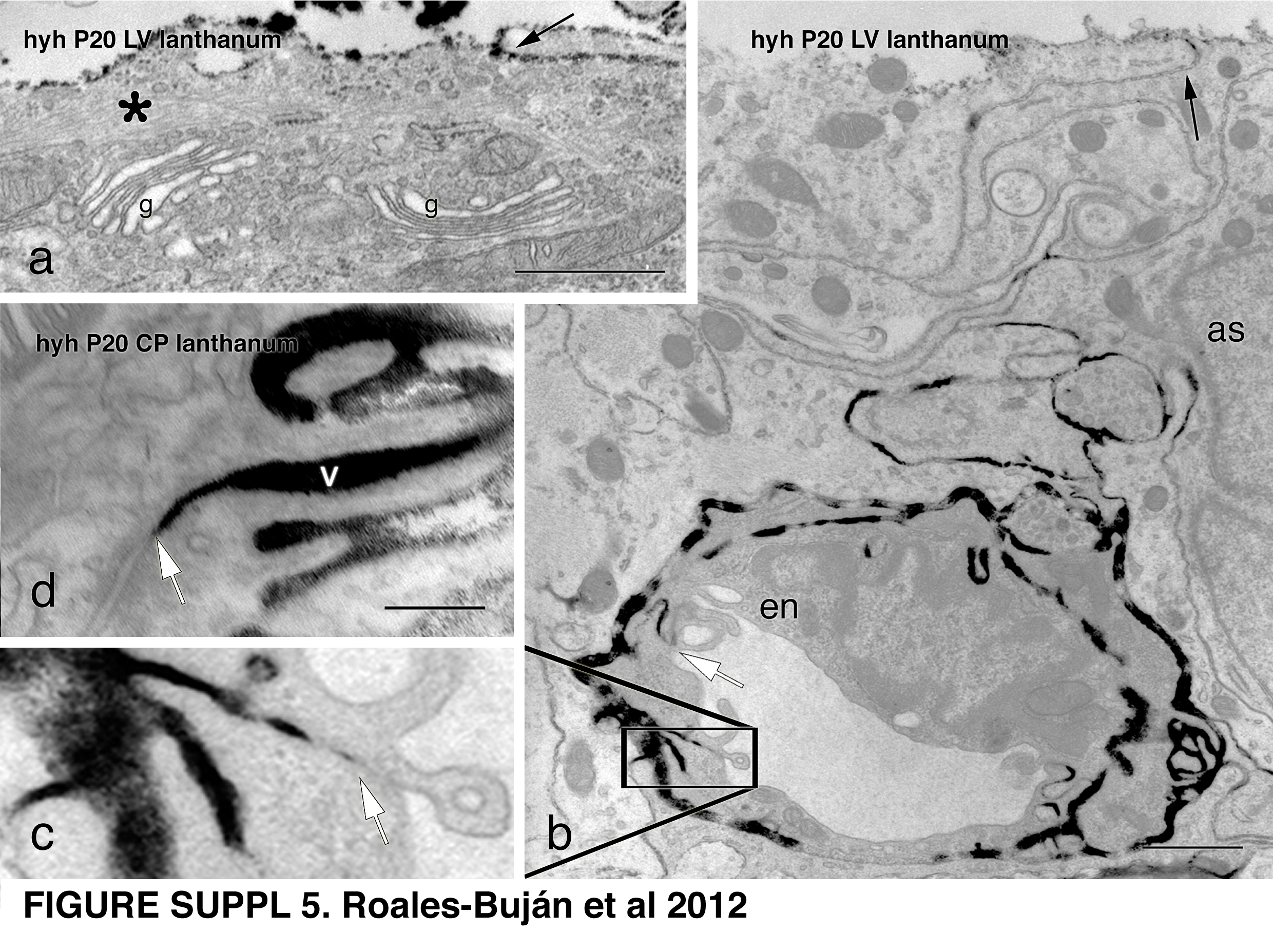

Supplement: Supplementary file 5 — Supplementary Figure 5. Ultrastructural detection of lanthanum nitrate applied into the lateral ventricle of a hyh mouse, at P20. (a-c; c is a detailed view of the area framed in b) Lanthanum penetrates from the ventricular lumen towards the brain parenchyma through the winding extracellular spaces of the astrocytic layer (as) lining the denuded ventricular surface (black arrows). Lanthanum reached the intercellular space of the neuropile and the pericapillary basement membrane, a transport pathway similar to that of the areas lined by ependyma. Tight junctions present in the endothelial cells (en; in b) prevent the extracellular progression of the tracer (white arrows; in b, c). (d) Tight junctions present in the choroid plexus ependyma also prevent the extracellular progression of the tracer (white arrow). Asterisk: intermediate filament bundles of astrocytes. Abbreviations: CP, Choroid plexus; g, Golgi apparatus dictiosomes; LV, lateral ventricle; V, intercellular space open to the ventricular lumen. Scale bars: a, 500 nm; b, 1 µm; b, 30 µm; d, 200 nm (TIFF 13478 kb) [file 401_2012_992_MOESM5_ESM.tiff]
