# Supplementary material for: Astrocytes acquire morphological and functional characteristics of ependymal cells following disruption of ependyma in hydrocephalus
Source: Acta Neuropathol. 2012 May 11;124(4):531–46. doi: 10.1007/s00401-012-0992-6 (PMC3444707; doi:10.1007/s00401-012-0992-6)
Supplement: Supplementary file 6 — Supplementary material 6 (DOCX 116 kb) [file 401_2012_992_MOESM6_ESM.docx]

**Supplementary material**

*Immunocytochemistry*

After perfusion of the mice with Bouin, brains were dissected out and immersed in the same fixative for 48 h. Tissue samples were embedded in paraffin and 10 µm thick serial sections were obtained. The secctions were hydrated and incubated in the firts antibodies. Appropriate secondary biotinylated antibodies and ExtrAvidin conjugated with peroxidase (Sigma) were subsequently used. 3,3´-diaminobenzidine tetrahydrochloride (DAB, Sigma) was used as an electron donor. Antibodies were diluted in 0.01 M phosphate saline buffer, pH 7.4, containing Triton-X100, bovine albumin, and normal sera. Incubations were performed at 22°C for 18 h for the primary antibodies and for 1 h for the secondary antibodies and ExtrAvidin. Some of the sections were stained with hematoxylin-eosin.

*Immunofluorescence*

Mice perfused with Bouin and paraformaldehyde and brains were removed and postfixed in the same solution for 24 h at 4ºC (paraformaldehyde) or for 48 h at room temperature (Bouin fixative). Bouin-fixed brains were embedded in paraffin and 10 µm frontal and sagittal serial sections were obtained and mounted in silanized slides. Brains fixed in paraformaldehyde were cryoprotected in 30% sucrose, and frozen sections (40 µm) were collected. Hydrated paraffin sections and frozen sections were incubated in the first antibodies followed of appropriate secondary antibodies: donkey anti-goat IgG, goat anti-rabbit IgG conjugated with Alexa Fluor 488, 568, 568, or 594 (1:2000 dilution; Molecular Probes, Carlsbad, CA) and goat anti-chicken IgY (ab6569, Abcam). Antibodies were diluted in 0.01 M phosphate buffer saline, pH 7.4, containing bovine serum albumin, appropriate normal sera, and 0.05% Triton-X100. Sections were studied under an epifluorescence Zeiss microscope and Leica TCS NT and Leica SP5 II confocal microscopes. Serial 1 µm thick planes and projections in Z-axis were obtained with confocal laser microscopy and processed with ImageJ software (NIH, USA). DAPI (Molecular Probes) nuclear staining was used in some sections for confocal and fluorescence microscopy.

Sections were mounted in Vectashield (Dako, Glostrup, Denmark) and whole mounts in PBS/glicerol (1:1), and inspected with an epifluorescence microscope equipped with the multidimensional acquisition software AxioVision Rel (version 4.6) of Zeiss (Jena, Germany) or confocal microscopy (see above). Omission of the primary antibody was used as a control test.

*Data analysis*

The following analyses were carried out. 1) The relative optic density of aquaporin 4 immunoreaction in ependymal cells and astrocytes compared to that of the perivascular endfeet astrocytes. 2) The total area occupied in frozen sections by the early endosomal compartment (EEA-1 immunoreactive) per ependymal cell or astrocyte. For each section, the sum of the area of all endosomes was calculated in the latero-medial ventricle wall of wt and hyh mice and divided per number of astrocytes or ependymal cells covering such surfaces. These analyses (1 and 2) were performed in frozen sections of the brain of 4 wt and 4 hyh mice at P20, processed for double immunofluorescence for aquaporin 4/GFAP or EEA1/GFAP with DAPI counterstaining. 3) The cell density of the astrocyte network at specific sites of the abnormal ventricles was estimated using whole mounts of ventricular walls of 4 hyh P20 mice processed for immunofluorescence for GFAP. 4) The degree of penetration of intraventricularly injected HRP into the brain parenchyma, at specific sites of the ventricular cavities, was estimated in paraffin sections of the brain of 4 hyh P8 mice immunostained using anti-HRP. All analyses (1-4) were made using micrographs from laser scanning microscopy and light microscopy using the software Visilog 6 (Noesis, Rolles, France). Statistical analyses were performed using SigmaStat (Systat Software, San Jose, CA) and KaleidaGraph (Synergy Software, Reading, PA).
